# (+)-(*S*)-*N*-[(1-Benzo­thio­phen-2-yl)methyl­idene]-1-(naphthalen-1-yl)ethyl­amine

**DOI:** 10.1107/S1600536813023611

**Published:** 2013-08-31

**Authors:** Guadalupe Hernández-Téllez, Oscar Portillo-Moreno, René Gutiérrez, Francisco J. Rios-Merino, Angel Mendoza

**Affiliations:** a Lab. Síntesis de Complejos, Facultad de Ciencias Químicas, Benemérita, Universidad Autónoma de Puebla, PO Box 1067, 72001 Puebla, Pue., Mexico; b Centro de Química, Instituto de Ciencias, Benemérita Universidad Autónoma de Puebla, 72570 Puebla, Pue., Mexico

## Abstract

In the title compound, C_21_H_17_NS, the C=N double bond shows an *E* conformation. The dihedral angle between the mean planes of the naphthyl residue and the benzo­thio­phene residue is 89.14 (6)°. The crystal packing is stabilized by inter­molecular C—H⋯π inter­actions, building a ribbon structure along the *a* axis.

## Related literature

For Schiff bases, see: García *et al.* (2011 ▶); Bernès *et al.* (2010 ▶); Jeon *et al.* (2005 ▶); Noyori (2005 ▶); Tanaka & Toda (2000 ▶).
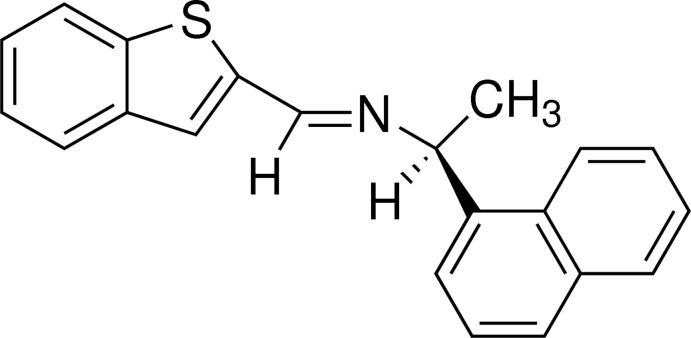


## Experimental

### 

#### Crystal data

C_21_H_17_NS
*M* *_r_* = 315.42
Orthorhombic, 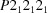

*a* = 5.6423 (3) Å
*b* = 8.0808 (4) Å
*c* = 36.3864 (19) Å
*V* = 1659.01 (15) Å^3^
*Z* = 4
Cu *K*α radiation
μ = 1.70 mm^−1^
*T* = 298 K
0.93 × 0.17 × 0.06 mm

#### Data collection

Oxford Diffraction Xcalibur (Atlas, Gemini) diffractometer
Absorption correction: multi-scan (*CrysAlis PRO*; Oxford Diffraction, 2006 ▶) *T*_min_ = 0.665, *T*_max_ = 1
8690 measured reflections
2844 independent reflections
2417 reflections with *I* > 2σ(*I*)
*R*_int_ = 0.053

#### Refinement

*R*[*F* ^2^ > 2σ(*F* ^2^)] = 0.040
*wR*(*F* ^2^) = 0.092
*S* = 1.01
2844 reflections
208 parameters
H-atom parameters constrained
Δρ_max_ = 0.25 e Å^−3^
Δρ_min_ = −0.18 e Å^−3^
Absolute structure: Flack parameter determined using 839 quotients [(I^+^)−(I^−^)]/[(I^+^)+(I^−^)] (Parsons & Flack (2004 ▶)
Absolute structure parameter: 0.021 (17)

### 

Data collection: *CrysAlis PRO* (Oxford Diffraction, 2006 ▶); cell refinement: *CrysAlis PRO*; data reduction: *CrysAlis PRO*; program(s) used to solve structure: *SHELXS97* (Sheldrick, 2008 ▶); program(s) used to refine structure: *SHELXL2013* (Sheldrick, 2008 ▶); molecular graphics: *SHELXL2013*; software used to prepare material for publication: *SHELXL2013*.

## Supplementary Material

Crystal structure: contains datablock(s) global, I. DOI: 10.1107/S1600536813023611/bt6929sup1.cif

Structure factors: contains datablock(s) I. DOI: 10.1107/S1600536813023611/bt6929Isup2.hkl

Supplementary material file. DOI: 10.1107/S1600536813023611/bt6929Isup3.cml

Additional supplementary materials:  crystallographic information; 3D view; checkCIF report

## Figures and Tables

**Table 1:** Hydrogen-bond geometry (Å, °) *Cg*1 and *Cg*2 are the centroids of the S1/C2/C3/C4/C9, C12/C13/C18/C19/C20/C21 rings, respectively.

*D*—H⋯*A*	*D*—H	H⋯*A*	*D*⋯*A*	*D*—H⋯*A*
C8—H8⋯*Cg*1^i^	0.93	2.73	3.491 (4)	139
C11—H11*B*⋯*Cg*2^ii^	0.96	2.59	3.724 (5)	149

Symmetry codes: (i) 
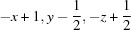
; (ii) 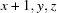.

**Fig. 1.**
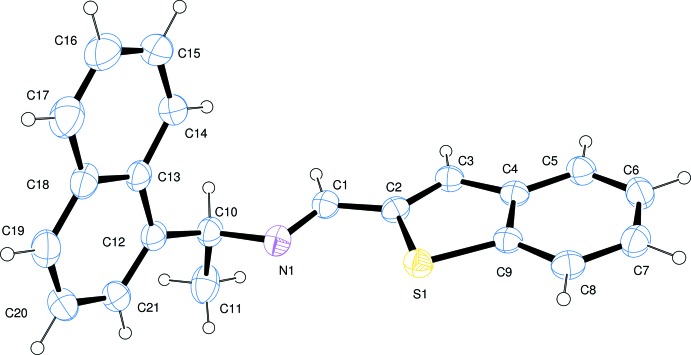
The molecular structure of title compound, with atom labels and 30% probability displacement ellipsoids for non-H atoms.

## supplementary crystallographic information

### 1. Comment

Schiff base compounds are widely studied and used, attracting much attention in both organic synthesis and metal ion complexation. Recently, we have focused our attention on the synthesis of chiral Schiff bases by using green techniques (García *et al.*, 2011; Bernès *et al.*, 2010). In continuation of this work, we synthesized the title compound using the solvent-free approach because the reactions occur under mild conditions and usually require easier workup procedures and simpler equipment. Other advantages of solvent-free reactions encompass cost saving, decreased reaction times along with reduced energy consumption, as well as increased safety (Jeon *et al.*, 2005; Noyori, 2005; Tanaka & Toda, 2000)

The C=N double bond shows an *E* configuration. The dihedral angle between the mean planes of the naphthyl residue and the benzothiophene residue is 90.86 (6)°. The crystal packing is stabilized by intermolecular C—H···π interactions (Table 2; cg1 is the centroid of the ring composed of S1, C2, C3 C4, and C9, cg2 is the centroid of the ring composed of C12, C13, C18, C19, C20, and C21).

### 2. Experimental

Under solvent-free conditions, (*S*)-(-)-(1-naphthyl)ethylamine (0.21 g, 1.2 mmol) and benzo[*b*]thiophene-2-carboxaldehyde (0.20 g, 1.2 mmol) were mixed at room temperature obtaining a white solid. The crude was recrystallized from CH_2_Cl_2_ affording colorless crystals of the title compound. Yield 94%; mp 125–127 °C. Analysis: [α]_D_^25^ = +318 (cL, CHCl_3_). FT—IR (KBr): 1621 cm-1 (C=N). ^1^H NMR (400 MHz, CDCl_3_/TMS) δ = 1.76, 1.78 (d, 3H, CHCH3,), 5.40, 5.42, 5.44, 5.45(q, ^1^H, CH), 7.32–7.89 (m, 12 H Ar), 8.20, 8.23 (d, 1H *cyclo* S), 8.54 (s, 1 H, HC=N). ^13^C NMR (100 MHz, CDCl_3_/TMS) δ = 24.20 (CCH_3_), 64.87 (CHCH_3_), 122.66 (Ar), 123.58 (Ar), 124.14 (Ar), 124.43(Ar), 125.36 (Ar), 125.66 (Ar), 125.90 (Ar), 127.47 (Ar), 127.62 (Ar), 128.91 (Ar), 130.58(Ar), 133.93 (Ar), 139.31 (Ar),140.57 (Ar), 143.16 (Ar), 153.61 (HC=N). MS—EI: *m/z*= 315 (*M*+).

### 3. Refinement

H atoms linked to C atoms were placed in geometrical idealized positions and refined as riding on their parent atoms, with C—H = 0.93–0.96 Å and with *U*_iso_(H) = 1.2 *U*_eq_(C) or 1.5 *U*_eq_(methyl C).

### Crystal data

**Table:**

C_21_H_17_NS	*F*(000) = 664
*M**_r_* = 315.42	*D*_x_ = 1.263 Mg m^−^^3^
Orthorhombic, *P*2_1_2_1_2_1_	Cu *K*α radiation, λ = 1.54184 Å
Hall symbol: P 2ac 2ab	Cell parameters from 2302 reflections
*a* = 5.6423 (3) Å	θ = 4.8–73.7°
*b* = 8.0808 (4) Å	µ = 1.70 mm^−^^1^
*c* = 36.3864 (19) Å	*T* = 298 K
*V* = 1659.01 (15) Å^3^	Plate, translucent colourless
*Z* = 4	0.93 × 0.17 × 0.06 mm

### Data collection

**Table:**

Oxford Diffraction Xcalibur (Atlas, Gemini) diffractometer	2844 independent reflections
Graphite monochromator	2417 reflections with *I* > 2σ(*I*)
Detector resolution: 10.5564 pixels mm^-1^	*R*_int_ = 0.053
ω scans	θ_max_ = 66.1°, θ_min_ = 4.9°
Absorption correction: multi-scan (*CrysAlis PRO*; Oxford Diffraction, 2006)	*h* = −6→6
*T*_min_ = 0.665, *T*_max_ = 1	*k* = −9→9
8690 measured reflections	*l* = −43→43

### Refinement

**Table:**

Refinement on *F*^2^	Hydrogen site location: inferred from neighbouring sites
Least-squares matrix: full	H-atom parameters constrained
*R*[*F*^2^ > 2σ(*F*^2^)] = 0.040	*w* = 1/[σ^2^(*F*_o_^2^) + (0.0363*P*)^2^] where *P* = (*F*_o_^2^ + 2*F*_c_^2^)/3
*wR*(*F*^2^) = 0.092	(Δ/σ)_max_ < 0.001
*S* = 1.01	Δρ_max_ = 0.25 e Å^−^^3^
2844 reflections	Δρ_min_ = −0.18 e Å^−^^3^
208 parameters	Absolute structure: Flack parameter determined using 839 quotients [(I+)-(I-)]/[(I+)+(I-)] (Parsons & Flack (2004)
0 restraints	Absolute structure parameter: 0.021 (17)

### Special details

**Table:**

Geometry. All e.s.d.'s (except the e.s.d. in the dihedral angle between two l.s. planes) are estimated using the full covariance matrix. The cell e.s.d.'s are taken into account individually in the estimation of e.s.d.'s in distances, angles and torsion angles; correlations between e.s.d.'s in cell parameters are only used when they are defined by crystal symmetry. An approximate (isotropic) treatment of cell e.s.d.'s is used for estimating e.s.d.'s involving l.s. planes.

### Fractional atomic coordinates and isotropic or equivalent isotropic displacement parameters (Å^2^)

**Table:**

	*x*	*y*	*z*	*U*_iso_*/*U*_eq_	
S1	0.66160 (15)	0.33795 (10)	0.20762 (2)	0.0504 (2)	
C13	0.8326 (7)	0.6220 (4)	0.07300 (9)	0.0526 (8)	
C5	0.8422 (8)	0.4832 (4)	0.30726 (10)	0.0559 (8)	
H5	0.9665	0.5439	0.3173	0.067*	
C9	0.6413 (6)	0.3627 (4)	0.25514 (9)	0.0453 (7)	
C3	0.9946 (6)	0.4998 (4)	0.24067 (10)	0.0492 (8)	
H3	1.1323	0.5599	0.2451	0.059*	
C4	0.8335 (6)	0.4527 (4)	0.26915 (10)	0.0467 (7)	
C2	0.9289 (5)	0.4490 (4)	0.20692 (11)	0.0485 (7)	
C1	1.0499 (6)	0.4771 (4)	0.17269 (10)	0.0506 (8)	
H1	1.182	0.546	0.1725	0.061*	
C6	0.6675 (8)	0.4233 (5)	0.32929 (11)	0.0612 (9)	
H6	0.6735	0.4435	0.3544	0.073*	
N1	0.9833 (5)	0.4116 (4)	0.14263 (9)	0.0557 (7)	
C12	0.9580 (6)	0.4687 (4)	0.07747 (10)	0.0524 (8)	
C8	0.4653 (6)	0.3017 (4)	0.27776 (11)	0.0553 (9)	
H8	0.3398	0.2412	0.2681	0.066*	
C10	1.1270 (7)	0.4460 (5)	0.10929 (10)	0.0563 (9)	
H10	1.2155	0.549	0.113	0.068*	
C19	0.6218 (8)	0.5002 (6)	0.02083 (11)	0.0696 (11)	
H19	0.51	0.5086	0.0021	0.083*	
C18	0.6615 (8)	0.6360 (5)	0.04455 (10)	0.0626 (9)	
C11	1.3029 (8)	0.3034 (6)	0.10488 (12)	0.0797 (13)	
H11A	1.4038	0.2983	0.1261	0.12*	
H11B	1.3975	0.3214	0.0833	0.12*	
H11C	1.2177	0.2012	0.1025	0.12*	
C17	0.5403 (10)	0.7867 (6)	0.04014 (15)	0.0848 (15)	
H17	0.4272	0.7966	0.0217	0.102*	
C7	0.4790 (7)	0.3319 (5)	0.31476 (11)	0.0611 (10)	
H7	0.362	0.2913	0.3303	0.073*	
C14	0.8719 (9)	0.7621 (5)	0.09573 (12)	0.0659 (11)	
H14	0.9816	0.7552	0.1147	0.079*	
C15	0.7523 (10)	0.9062 (6)	0.09026 (14)	0.0851 (15)	
H15	0.7825	0.997	0.1052	0.102*	
C20	0.7450 (8)	0.3587 (6)	0.02515 (12)	0.0700 (11)	
H20	0.7182	0.2704	0.0093	0.084*	
C16	0.5852 (10)	0.9180 (6)	0.06237 (15)	0.0950 (18)	
H16	0.5034	1.0167	0.0589	0.114*	
C21	0.9123 (7)	0.3431 (5)	0.05316 (10)	0.0610 (9)	
H21	0.9956	0.2443	0.0554	0.073*	

### Atomic displacement parameters (Å^2^)

**Table:**

	*U*^11^	*U*^22^	*U*^33^	*U*^12^	*U*^13^	*U*^23^
S1	0.0487 (4)	0.0531 (4)	0.0493 (4)	−0.0025 (4)	−0.0075 (4)	−0.0035 (4)
C13	0.0587 (19)	0.0561 (19)	0.0431 (17)	0.0004 (18)	0.0125 (17)	0.0075 (14)
C5	0.059 (2)	0.0567 (19)	0.0522 (19)	−0.002 (2)	−0.0078 (19)	−0.0037 (16)
C9	0.0447 (16)	0.0411 (14)	0.0501 (17)	0.0052 (15)	−0.0051 (16)	0.0011 (13)
C3	0.0460 (19)	0.0482 (17)	0.054 (2)	−0.0028 (15)	−0.0055 (16)	−0.0030 (15)
C4	0.0446 (17)	0.0415 (14)	0.0541 (18)	0.0071 (16)	−0.0046 (18)	−0.0002 (14)
C2	0.0472 (17)	0.0431 (16)	0.0552 (19)	0.0040 (13)	−0.0013 (17)	0.0042 (16)
C1	0.0484 (18)	0.0518 (18)	0.051 (2)	0.0041 (15)	−0.0027 (17)	0.0030 (16)
C6	0.070 (2)	0.065 (2)	0.0485 (19)	0.005 (2)	0.002 (2)	−0.0040 (18)
N1	0.0539 (17)	0.0614 (17)	0.0517 (18)	0.0019 (15)	0.0030 (14)	0.0063 (15)
C12	0.055 (2)	0.0558 (18)	0.0461 (19)	0.0009 (17)	0.0109 (17)	0.0041 (16)
C8	0.0486 (19)	0.0497 (19)	0.068 (2)	−0.0031 (16)	−0.0031 (18)	−0.0008 (17)
C10	0.058 (2)	0.061 (2)	0.0497 (19)	−0.0035 (18)	0.0073 (18)	0.0045 (16)
C19	0.077 (3)	0.082 (3)	0.049 (2)	−0.005 (2)	−0.004 (2)	0.006 (2)
C18	0.067 (2)	0.070 (2)	0.0502 (19)	0.007 (2)	0.008 (2)	0.0127 (18)
C11	0.070 (3)	0.103 (4)	0.066 (3)	0.022 (3)	0.012 (2)	0.017 (3)
C17	0.093 (3)	0.090 (3)	0.072 (3)	0.025 (3)	−0.001 (3)	0.015 (3)
C7	0.061 (2)	0.060 (2)	0.062 (2)	0.003 (2)	0.0126 (19)	0.009 (2)
C14	0.085 (3)	0.059 (2)	0.054 (2)	0.003 (2)	0.010 (2)	0.0002 (17)
C15	0.127 (4)	0.060 (2)	0.069 (3)	0.007 (3)	0.022 (3)	0.000 (2)
C20	0.087 (3)	0.070 (3)	0.053 (2)	−0.009 (2)	0.003 (2)	−0.009 (2)
C16	0.128 (5)	0.073 (3)	0.084 (4)	0.039 (3)	0.020 (3)	0.016 (3)
C21	0.073 (2)	0.0584 (19)	0.052 (2)	0.0054 (18)	0.0099 (18)	−0.0017 (19)

### Geometric parameters (Å, º)

**Table:**

S1—C9	1.744 (3)	C8—H8	0.93
S1—C2	1.755 (3)	C10—C11	1.529 (6)
C13—C14	1.419 (5)	C10—H10	0.98
C13—C18	1.420 (6)	C19—C20	1.347 (6)
C13—C12	1.436 (5)	C19—C18	1.414 (6)
C5—C6	1.359 (6)	C19—H19	0.93
C5—C4	1.409 (5)	C18—C17	1.406 (6)
C5—H5	0.93	C11—H11A	0.96
C9—C8	1.380 (5)	C11—H11B	0.96
C9—C4	1.402 (5)	C11—H11C	0.96
C3—C2	1.347 (5)	C17—C16	1.358 (7)
C3—C4	1.430 (5)	C17—H17	0.93
C3—H3	0.93	C7—H7	0.93
C2—C1	1.438 (5)	C14—C15	1.361 (6)
C1—N1	1.272 (5)	C14—H14	0.93
C1—H1	0.93	C15—C16	1.388 (7)
C6—C7	1.399 (6)	C15—H15	0.93
C6—H6	0.93	C20—C21	1.395 (6)
N1—C10	1.485 (5)	C20—H20	0.93
C12—C21	1.371 (6)	C16—H16	0.93
C12—C10	1.511 (6)	C21—H21	0.93
C8—C7	1.371 (6)		
			
C9—S1—C2	90.70 (18)	C12—C10—H10	108.9
C14—C13—C18	117.9 (4)	C11—C10—H10	108.9
C14—C13—C12	123.0 (4)	C20—C19—C18	120.4 (4)
C18—C13—C12	119.1 (3)	C20—C19—H19	119.8
C6—C5—C4	119.5 (4)	C18—C19—H19	119.8
C6—C5—H5	120.2	C17—C18—C19	121.7 (4)
C4—C5—H5	120.2	C17—C18—C13	118.9 (4)
C8—C9—C4	121.6 (3)	C19—C18—C13	119.4 (4)
C8—C9—S1	126.7 (3)	C10—C11—H11A	109.5
C4—C9—S1	111.7 (3)	C10—C11—H11B	109.5
C2—C3—C4	113.9 (3)	H11A—C11—H11B	109.5
C2—C3—H3	123.1	C10—C11—H11C	109.5
C4—C3—H3	123.1	H11A—C11—H11C	109.5
C9—C4—C5	118.4 (4)	H11B—C11—H11C	109.5
C9—C4—C3	111.5 (3)	C16—C17—C18	121.2 (5)
C5—C4—C3	130.1 (4)	C16—C17—H17	119.4
C3—C2—C1	127.6 (3)	C18—C17—H17	119.4
C3—C2—S1	112.3 (3)	C8—C7—C6	120.5 (4)
C1—C2—S1	120.1 (3)	C8—C7—H7	119.7
N1—C1—C2	122.6 (3)	C6—C7—H7	119.7
N1—C1—H1	118.7	C15—C14—C13	121.3 (5)
C2—C1—H1	118.7	C15—C14—H14	119.3
C5—C6—C7	121.1 (4)	C13—C14—H14	119.3
C5—C6—H6	119.5	C14—C15—C16	120.2 (5)
C7—C6—H6	119.5	C14—C15—H15	119.9
C1—N1—C10	117.6 (3)	C16—C15—H15	119.9
C21—C12—C13	118.2 (3)	C19—C20—C21	120.7 (4)
C21—C12—C10	121.5 (3)	C19—C20—H20	119.6
C13—C12—C10	120.2 (3)	C21—C20—H20	119.6
C7—C8—C9	118.8 (4)	C17—C16—C15	120.6 (4)
C7—C8—H8	120.6	C17—C16—H16	119.7
C9—C8—H8	120.6	C15—C16—H16	119.7
N1—C10—C12	107.7 (3)	C12—C21—C20	122.1 (4)
N1—C10—C11	107.4 (3)	C12—C21—H21	118.9
C12—C10—C11	114.9 (3)	C20—C21—H21	118.9
N1—C10—H10	108.9		
			
C2—S1—C9—C8	−178.8 (3)	C1—N1—C10—C11	95.0 (4)
C2—S1—C9—C4	0.0 (2)	C21—C12—C10—N1	−99.0 (4)
C8—C9—C4—C5	−1.2 (5)	C13—C12—C10—N1	78.2 (4)
S1—C9—C4—C5	179.9 (3)	C21—C12—C10—C11	20.6 (5)
C8—C9—C4—C3	178.8 (3)	C13—C12—C10—C11	−162.1 (3)
S1—C9—C4—C3	−0.1 (3)	C20—C19—C18—C17	178.3 (4)
C6—C5—C4—C9	0.9 (5)	C20—C19—C18—C13	0.0 (6)
C6—C5—C4—C3	−179.1 (3)	C14—C13—C18—C17	0.2 (6)
C2—C3—C4—C9	0.2 (4)	C12—C13—C18—C17	−179.6 (4)
C2—C3—C4—C5	−179.9 (4)	C14—C13—C18—C19	178.5 (4)
C4—C3—C2—C1	179.4 (3)	C12—C13—C18—C19	−1.2 (6)
C4—C3—C2—S1	−0.2 (4)	C19—C18—C17—C16	−177.9 (5)
C9—S1—C2—C3	0.1 (3)	C13—C18—C17—C16	0.4 (7)
C9—S1—C2—C1	−179.5 (3)	C9—C8—C7—C6	0.2 (6)
C3—C2—C1—N1	173.8 (4)	C5—C6—C7—C8	−0.5 (6)
S1—C2—C1—N1	−6.7 (5)	C18—C13—C14—C15	−0.9 (6)
C4—C5—C6—C7	0.0 (6)	C12—C13—C14—C15	178.9 (4)
C2—C1—N1—C10	−178.2 (3)	C13—C14—C15—C16	1.0 (7)
C14—C13—C12—C21	−177.7 (4)	C18—C19—C20—C21	0.4 (7)
C18—C13—C12—C21	2.0 (5)	C18—C17—C16—C15	−0.3 (8)
C14—C13—C12—C10	5.0 (5)	C14—C15—C16—C17	−0.4 (8)
C18—C13—C12—C10	−175.3 (3)	C13—C12—C21—C20	−1.7 (6)
C4—C9—C8—C7	0.7 (5)	C10—C12—C21—C20	175.6 (3)
S1—C9—C8—C7	179.4 (3)	C19—C20—C21—C12	0.4 (6)
C1—N1—C10—C12	−140.7 (3)		

### Hydrogen-bond geometry (Å, º)

*Cg*1 and *Cg*2 are the centroids of the S1/C2/C3/C4/C9, C12/C13/C18/C19/C20/C21 rings, respectively.

**Table:**

*D*—H···*A*	*D*—H	H···*A*	*D*···*A*	*D*—H···*A*
C8—H8···*Cg*1^i^	0.93	2.73	3.491 (4)	139
C11—H11*B*···*Cg*2^ii^	0.96	2.59	3.724 (5)	149

Symmetry codes: (i) −*x*+1, *y*−1/2, −*z*+1/2; (ii) *x*+1, *y*, *z*.
